# CCDC97 is a Promising Predictor in Cancer Diagnosis: A Pan Cancer Analysis

**DOI:** 10.1155/ancp/2230769

**Published:** 2026-01-08

**Authors:** Changye Yang, Ruirui Yang, Rundong Wang, Guo Ma, Lixin Hua

**Affiliations:** ^1^ Department of Clinical Medicine, Affiliated Huishan Hospital of Xinglin College, Nantong University, No.2 Zhanqian North Road, Wuxi, 214000, Jiangsu, China, ntu.edu.cn; ^2^ Ministry of Science and Education, Affiliated Huishan Hospital of Xinglin College, Nantong University, No.2 Zhanqian North Road, Wuxi, 214000, Jiangsu, China, ntu.edu.cn; ^3^ School of Clinical and Basic Medicine, Shandong First Medical University, Jinan, China, sdfmu.edu.cn; ^4^ School of Pharmacy, National Key Laboratory of Advanced Drug Formulations for Overcoming Delivery Barriers, Fudan University, Shanghai, China, fudan.edu.cn; ^5^ Department of General Surgery, Affiliated Huishan Hospital of Xinglin College, Wuxi Huishan District People’s Hospital, Nantong University, Wuxi, China, ntu.edu.cn

**Keywords:** CCDC97, ceRNA network, DNA methylation, immune cell infiltration, pan cancer

## Abstract

**Background:**

Coiled‐coil domain‐containing 97 (CCDC97) can bind to the splicing factor SF3B complex to participate significantly in pre‐mRNA splicing. However, there may be a risk of cancer development in the case of disrupted mechanism, necessitating further in‐depth investigation of the underlying mechanism.

**Methods:**

The present study was conducted by searching for various databases to implement a comprehensive cancer analysis of CCDC97. Furthermore, a competitive endogenous RNA (ceRNA) network of CCDC97 was established, which was further integrated with the reported oncogene mechanisms in the CCDC family to explain the potential reasons for its abnormal expression.

**Results:**

CCDC97 is highly expressed in various cancers and closely associated with poor prognosis. CCDC97 was also strongly associated with the infiltration of various immune cells and multiple immune checkpoints in the tumor immune microenvironment. RT‐qPCR showed that CCDC97 was highly expressed in hepatocellular carcinoma (HCC) tissue samples. At the single‐cell level, CCDC97 exhibited an intimate association with depleted T cells in HCC tissues.

**Conclusion:**

In cancers, particularly in HCC CCDC97 can serve as a promising marker for immunotherapy and prognosis in cancers, particularly in HCC. More experimental verification is needed in the future research.

## 1. Introduction

Nowadays, one of the urgent threats that humanity faces is the risk of various malignant tumors. Critically, numerous attempts to conquer tumors have never stopped in worldwide, despite numerous difficulties. Among these, the coiled‐coil domain‐containing (CCDC) family is currently a research hot spot.

The protein family containing the CCDC is encoded by approximately 180 genes, and about 80 genes were associated with cancer in early studies. Among them, the functions and/or mechanisms of over 30 genes in tumors have been elucidated, yet with the lack of more in‐depth investigation on the remaining members in tumors [1]. For example, the activation of Snail by CCDC12, which is overexpressed in colorectal cancer (CRC), facilitates the invasion and pathogenesis of CRC glands [2]. In a mouse model of hepatocellular carcinoma (HCC), CCDC50 undergoes splicing of SRSF3, leading to the formation of CCDC50S.

The occurrence of this splicing event may activate the Ras signaling to enhance the proliferation of HCC [3]. Furthermore, downregulated miR‐338‐3p expression may upregulate CCDC56, triggering DRP1 phosphorylation‐mediated mitochondrial damage, in turn resulting in significantly increased in glucose metabolism and non‐small cell lung cancer deterioration [4]. Meanwhile, CCDC106 may inhibit p21 transcription and trigger AKT phosphorylation. thereby accelerating cancer cell in ovarian and non‐small cell lung tumors by inducing the expression of cyclin B1 and A2 [5, 6]. BRCA1‐related protein 2 is targeted by CCDC178, leading to its degradation. The activation of this ERK pathway can promote the metastasis and treatment resistance of HCC [7]. Altogether, in the presence of abnormal expression of genes from the CCDC family, a number of intricate processes and signaling pathways may be involved and contribute significantly to the growth, invasion, and metastasis of malignancies.

In this study, we reported a very promising gene CCDC97 in the CCDC family. Our elucidation of CCDC97 in the whole cancer sample compared to normal tissues, supported the role of high CCDC97 expression in causing poor prognosis of HCC. In addition, in order to explore the possible underlying mechanism of CCDC97 in tumor growth, we also studied the phosphorylation modification, promoter methylation, gene mutation, immune infiltration, single‐cell expression characteristics, and functional enrichment of CCDC97.

Findings in our study unveil that CCDC97 has similar mechanisms to other aberrantly expressed genes in this family, demonstrating its predictive value in various tumors, especially in HCC. Our investigation may provide potential insights into specific mechanisms, which are valuable for future research.

## 2. Methods

In this study, CCDC97 expression in cancers were comprehensively and systematically analyzed at the gene, protein, and single‐cell levels using six open‐source online databases. With data preprocessing conducted by using the FineDataLink softwar, further data processing was continued with corresponding methods detailed as given later.

### 2.1. Gene Expression Analysis

Initially, CCDC97 expression levels in tumor and nontumor tissues in various TCGA malignancies were assessed by TIMER2.0 (http://timer.cistrome.org/) [8]. Furthermore, GEPIA2 (http://gepia2.cancer-pku.cn/) was utilized to evaluate the differential expression of CCDC97 among tumor and nontumor tissues in TCGA tumors. The criteria were set to: Log2 (fold change) cutoff = 1, *p*‐value cutoff = 0.01, and “Match TCGA normal and GTEx data” of the parameters [9]. Meanwhile, the expression of CCDC97 at various clinical phases in TCGA cancers was realized using GEPIA2. Violin or box plots were created from the expression data transformed using the log2 [TPM (transcripts per million) + 1] approach.

UALCAN, an Omics analysis tool UALCAN (http://ualcan.path.uab.edu/index.html), and the Clinical Proteomic Tumor examination Consortium (CPTAC) datasets were utilized for examining protein expressions [10]. Our main goal was to investigate the difference in CCDC97 protein expression levels between tumor and non‐tumor tissues, with a particular focus on the amount of phosphorylated proteins.

### 2.2. Survival Analysis

The overall survival (OS) and disease‐free survival (DFS) in different types of malignancies were analyzed based on the assessment of CCDC97 expression using the “Survival Analysis” module in GEPIA2. A threshold of 50% was employed to distinguish between the high‐ and low‐expression groups. In addition, the hypothesis test was performed by using the log‐rank test.

### 2.3. Genetic Alteration Analysis

The cBioPortal database (https://www.cbioportal.org/) [11] was employed to examine the genetic modification traits of CCDC97. The “TCGA Pan‐Cancer Atlas Studies” consisted of 32 studies, including a total of 10,967 samples. The studies included the “Oncoprint,” “Cancer Type Summary,” and “Mutations” modules, aiming at offering an in‐depth information regarding the genetic alterations and locations of CCDC97 mutations. Furthermore, the association between probable copy number alterations (CNAs) of CCDC97 and its expression in cancer was determined by the “Plots” module. Simultaneously, the OS, DFS, progression‐free survival (PFS), and disease‐specific survival (DSS) of TCGA cancer patients with and without a CCDC97 genetic mutation, the “Comparison/Survival” module was utilized. The “Combined Study (10,967 samples)” was carried out by using the “Cancer Type (TCGA, PanCancer Atlas)” specifically. In addition, Kaplan–Meier plots were created and log‐rank *p*‐values were calculated as well. Moreover, the component was utilized for the examination of genetic alterations.

### 2.4. DNA Methylation Analysis

In order to ascertain what differences that exist between tumors and normal tissues, this study continued to investigate the DNA methylation levels of CCDC97 promoter in many different cancers on the basis of the UALCAN dataset (http://ualcan.path.uab.edu/index.html).

The analysis of DNA methylation mode survival was performed with the help of a web‐based service known as MethSurv (https://Biit.cs.ut.ee/MethSurv/) [12]. “Gene visualization” in MethSurv database can offer the DNA methylation map for CCDC97. SangerBox 3.0’s “Pan Cancer Analysis mRNA Modification” module (http://sangerbox.com/) biological data analysis could also be provided by analyzing Spearman’s rho value and statistical significance [13].

### 2.5. CCDC97 Expression and Immune Correlation Analysis

The correlation between immunological checkpoints and CCDC97 expression was further investigated by applying SangerBox3.0. The “Gene_Corr” module of TIMER2.0 was used to establish the relationship between mismatch repairs (MMRs) and CCDC97 expression. Furthermore, the associations between CCDC97 expression and microsatellite instability (MSI), tumor mutational burden (TMB), and neoantigen (NEO) were identified based on the SangerBox3.0’s “Cloud Platform [Legacy] – gene – gene‐immune analysis” module. SangerBox3.0 provided six methods for forecasting the immunological properties of CCDC97, facilitating a thorough investigation into the correlation between immune cell infiltration and CCDC97 communication. The statistical significance and Spearman’s rho values were essential for all the aforementioned analyses.

### 2.6. Single‐Cell Analysis and Drug Sensitivity Analysis

In this study, CCDC97 expression was characterized in the tumor microenvironment (TME) across multiple types of cancer using the Tumor Immune Single Cell Hub 2 (TISCH2) platform (http://tisch.comp-genomics.org/home/) [14], a single‐cell RNA sequencing database focusing on the TME that can provide extensive single‐cell cell‐type annotation.

Meanwhile, drug sensitivity analysis and gene expression analysis were also realized by using GSCALite (http://bioinfo.life.hust.edu.cn/web/GSCALite/) [15].

### 2.7. CCDC97‐Related Gene Enrichment Analysis

STRING (https://string-db.org/) [16] was employed to acquire proteins that interacted with CCDC97. The “Similar Gene Detection” module in GEPIA2 was used to retrieve the top‐100 TCGA–derived genes related to CCDC97. This correlation would provide evidence to determine potential connection across different forms of cancer. The correlation analysis was conducted using GEPIA2, and the heatmap data for genes associated with CCDC97 was obtained using TIME2.0. Ultimately, the pathway and function of the CCDC97‐correlated gene were examined by utilizing Kyoto Encyclopedia of Genes and Genomes (KEGG) and Gene Ontology (GO) on WebGestalt (http://www.webgestalt.org) [17]. The essential settings included “*Homo sapiens*,” “Over‐Representation Analysis (ORA),” “Gene Ontology,” and “Biological Process” with the “Genome” selected as the reference set.

### 2.8. Construction of Competitive Endogenous RNA (ceRNA) Network

The present study also established a regulatory network incorporating CCDC97, microRNA (miRNA), and long noncoding RNA (lncRNA). Three online prediction databases were used, that is, miRWalk (http://mirwalk.umm.uni-heidelberg.de/), miRDB (http://mirdb.org/miRDB/), and TargetScan (https://www.targetscan.org/vert_80/) [18–20] were used to predict miRNAs targeted by CCDC97. The target miRNAs were by intersecting the three locations. The relationship between lncRNA and miRNA, as well as the significance of miRNA and CCDC97 (lncRNA), were analyzed using starBase2.0 (https://starbase.sysu.edu.cn/) [21]. The screening criteria involved the usage of mammals, (i.e., humans), with the hg19 genome. The criterion required a rigorous stringency level of at least five for the CLIP‐Data, which might include or exclude the Degradome‐Data.

### 2.9. Statistical Analysis

For bioinformatics analysis, the entire data set was filtered by removing missing and duplicated data. All statistical analysis and visualization were conducted using the R software (version 3.6.3).

The correlation analysis was unified as Spearman correlation analysis. The inter‐group comparison of continuous variables was achieved by using the *t*‐test and and was considered statistically significant.

## 3. Results

### 3.1. The Characteristic of CCDC97 Expression in Pan Cancer

Initially, the overall expression pattern of CCDC97 across different types of cancer were analyzed and described by utilizing the TGCA database. CCDC97 expression was significantly upregulated in many malignancies including BRCA, CHOL, ESCA, GBM, head and neck squamous cell carcinoma (HNSC), KIRC, LIHC, LUSC, and STAD, along with 33 other types of cancer (Figure 1A). In contrast to the normal control group in the thyroid carcinoma (THCA), CCDC97 expression was considerably reduced in tumor tissues (Figure 1A). In certain tumors LAML, low‐grade glioma (LGG), PAAD, and THYM, CCDC97 expression levels were significantly higher in tumor tissues compared to normal tissues (Figure 1B), yet without obvious differences in other malignancies (Figure S1A). However, this study failed to compare with certain cancers given the absence of normal tissues in the TCGA database. According to the protein expression in the CPTAC database, CCDC97 protein levels were significantly higher in COAD, GBM, HCC, KIRC, and LUSC than nontumor tissues (Figure 1C). In addition, CCDC97 expression exhibited strong correlation with the pathological stage strongly correlated in adrenal cortical carcinoma (ACC) (*p* = 0.0135), KIRC (*p* = 0.0192), LIHC (*p* = 0.023), PAAD (*p* = 0.034), and READ (*p* = 0.0256) (Figure 1D and Figure S1B).

Figure 1The level of CCDC97 gene expression across different types of tumors and stages of disease. (A) CCDC97 expression level in various types of cancer.  ^∗^ *p* < 0.05;  ^∗∗^ *p* < 0.01;  ^∗∗∗^ *p* < 0.001. (B) The GTEx database provided corresponding normal tissues as control samples for the types of LAML, LGG, PAAD, and THYM in the TCGA investigation.  ^∗^ *p* < 0.01. (C) The total protein expression of CCDC97 in normal tissue and primary tumor (blue is nomal and red is primary tumor).  ^∗∗^ *p* < 0.01;  ^∗∗∗^ *p* < 0.001. (D) The CCDC97 gene’s expression levels were examined according to the four pathogenic phases (Stage I–IV). The data was converted to a logarithmic scale by taking the base 2 logarithm of the sum of TPM and 1.

**Figure:**
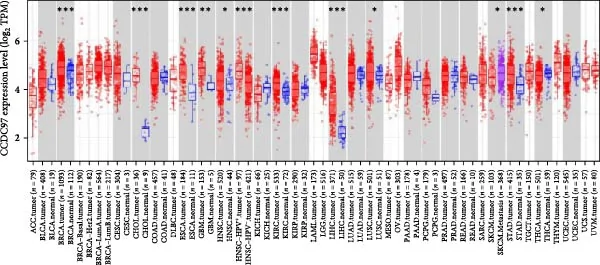
(A)

**Figure:**
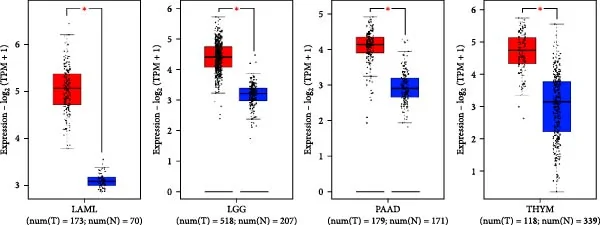
(B)

**Figure:**
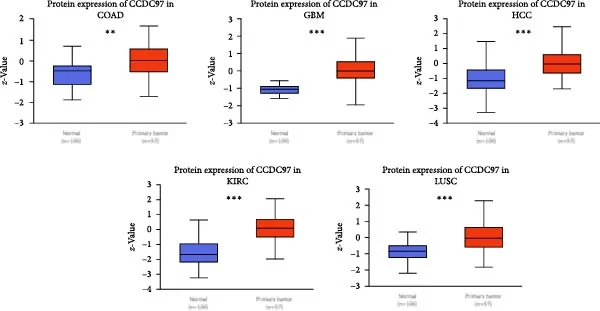
(C)

**Figure:**
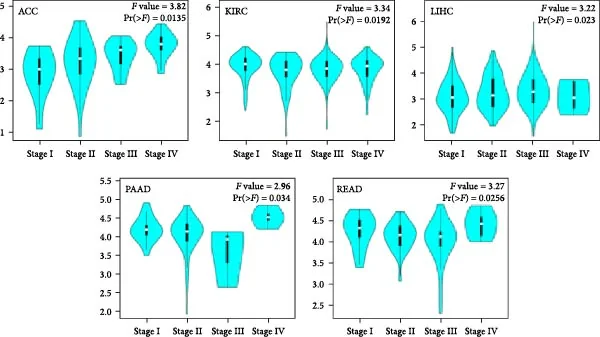
(D)

### 3.2. The Survival Prognosis Value of CCDC97 in Pan Cancer

The Kaplan–Meier estimation was employed to evaluate the prognostic significance of CCDC97. Consequently, high CCDC97 expression had a significant correlation with unfavorable OS in patients with ACC (HR = 3.1, log‐rank *p* = 0.0061), LGG (HR = 2, log‐rank *p* = 0.00027), and LIHC (HR = 1.6, log‐rank *p* = 0.014; Figure 2A). A decreased CCDC97 gene expression was associated to a worse KIRC prognosis (HR = 0.68, log‐rank *p* = 0.014; Figure 2A). Additionally, based on DFS analysis, ACC (HR = 2.8, log‐rank *p* = 0.0038), bladder cancer (BLCA; HR = 1.4, log‐rank *p* = 0.0038), LGG (HR = 1.5, log‐rank *p* = 0.0083), and LIHC (HR = 1.4, log‐rank *p* = 0.029) cases from TCGA were significantly linked to unfavorable prognosis based on DFS analysis (Figure 2B).

Figure 2Investigating the correlation between tumor survival prognosis in TCGA and the expression levels of the CCDC97 gene. (A) Analysis of overall survival and (B) analysis of disease‐free survival in various cancers in TCGA based on CCDC97 gene expression. The survival map and Kaplan–Meier curves displaying favorable outcomes are provided.

**Figure:**
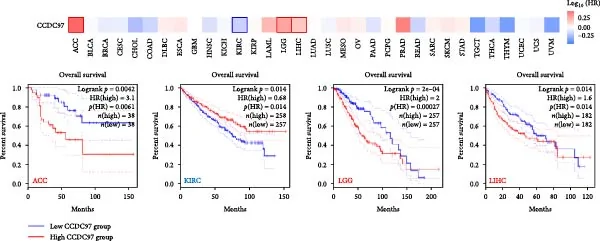
(A)

**Figure:**
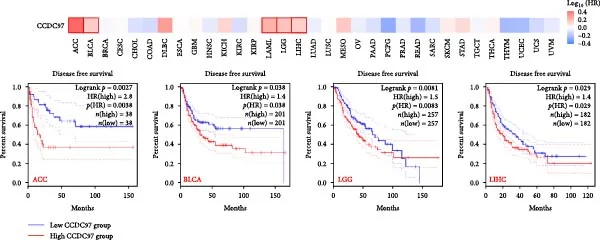
(B)

### 3.3. Genetic Analysis of CCDC97 in Pan Cancer

A spectrum of tumor samples from the TCGA cohorts was further explored to confirm the presence of CCDC97 genetic variant. As shown in Figure 3A, the highest rate of change (>7%) in CCDC97 was observed in individuals with uterine malignancies featured primarily by “amplification.” Approximately 3.5% of the cases of sarcoma cancer exhibited copy number “amplification” CNA, which was the most prevalent type (Figure 3A). Figure 3B also presents additional details regarding the categories, positions, and quantity of instances linked to CCDC97 genetic mutation. A total of 72 mutation sites were found between amino acids 0 and 343, including 59 missenses, six truncations, one inframe mutation, one splice mutation, and five fusions (Figure 3B). The R148 mutation, identified in two cases of SKCM and two cases of STAD (Figure 3B), induces a missense mutation in CCDC97 gene, leading to the replacement of arginine (R) with either cystine (C), serine (S), or histidine (H) at position 148 of the CCDC97 protein. The 3D configuration of the CCDC97 protein, as shown in Figure 3C, displays the existence of the R148 locationC. Figure 3D depicts the link between CCDC97 gene expression across different cancer types and the projected CNA. In addition, compared to the unmodified group, gene modifications of LINC01114, LINC01159, PANTR1, MRPS9‐AS1, MRPS9‐AS2, PLGLA, LINC02946, DELEC1, GGTA1, and ITPK1‐AS1 were more widespread in the group with CCDC97 alteration (Figure 3E).

Figure 3Analysis of CCDC97 mutation patterns across various cancers in the TCGA dataset. (A, B) The frequency of alterations is given according to the kind and site of the mutation. (C) In the 3D structure of CCDC97, the location of the mutation that has the highest proportion of modifications (R148S/C/H). (D) The putative CNA of CCDC97 and its expression in tumors are correlated. (E) The corresponding frequency of gene modification in groups with and without CCDC97 alteration. (F) Investigations of the possible relationship between BRCA, SARC, UCEC, and UCS progression‐free survival and mutation status.

**Figure:**
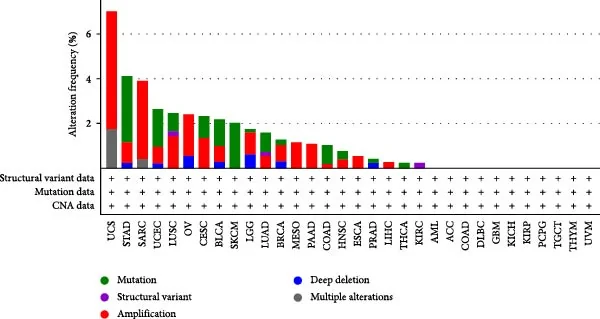
(A)

**Figure:**
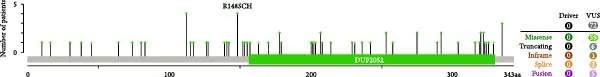
(B)

**Figure:**
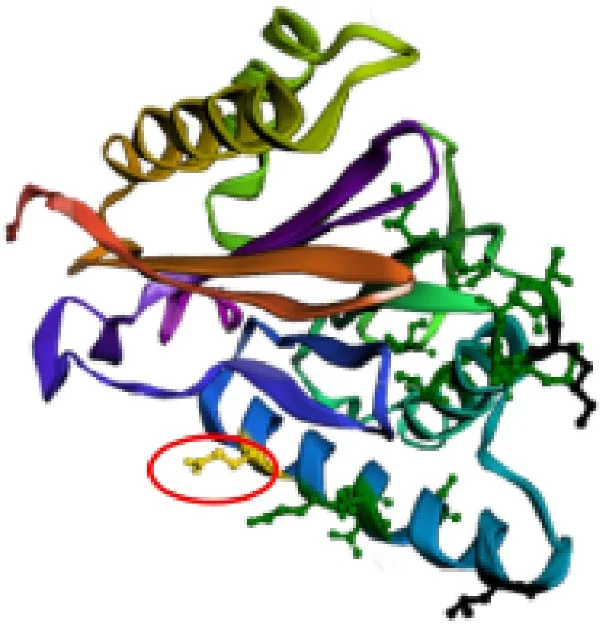
(C)

**Figure:**
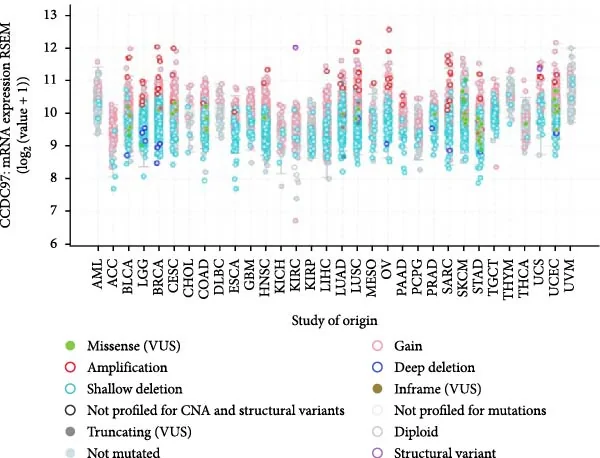
(D)

**Figure:**
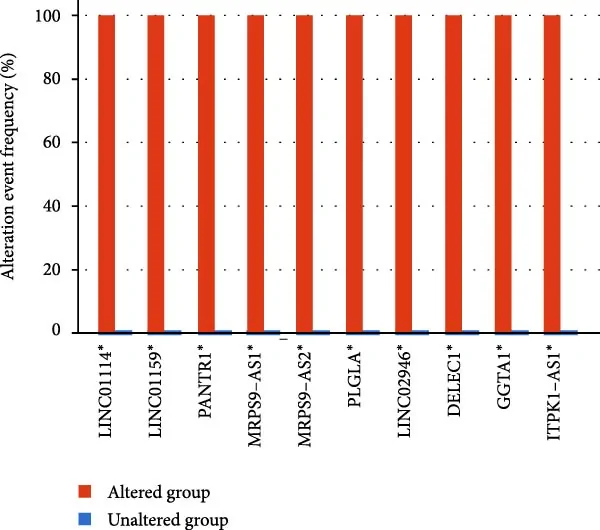
(E)

**Figure:**
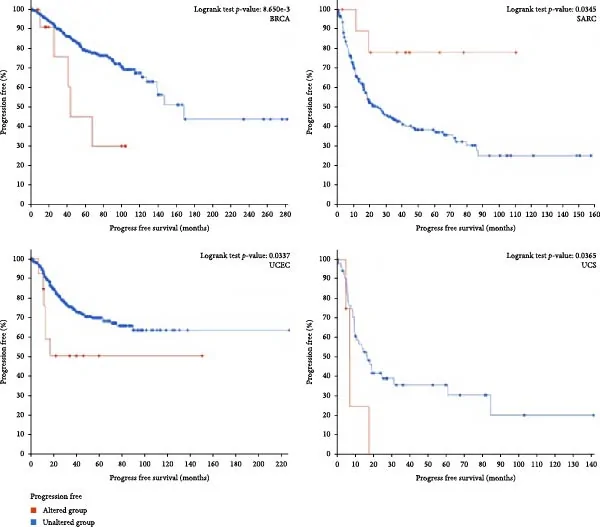
(F)

This study continued to examine the relationship between CCDC97 genetic alteration and the survival of cancer patients. Noticeably, there was a significant rise in the duration of disease‐free progression in patients without CCDC97 gene mutation in cases of BRCA, uterine corpus endometrial carcinoma (UCEC), and UCS (*p* = 1.604e−3, *p* = 8.650e−3, and *p* = 0.0365, respectively). Nevertheless, there was no discernible improvement regarding the unmodified SRAC cases (*p* = 0.0345; Figure 3F). Meanwhile, there was no statistically significant difference in DFS in both the modified and unmodified groups, including BRCA, SARC, and UCEC (*p* = 0.0531, *p* = 0.163, and *p* = 0.0633, respectively). Due to insufficient data, it was unable to determine the DFS of UCS cases (Figure S2A). Enhanced survival rates unique to the disease were only detected in UCEC cases without modification (*p* = 1.604e−3), but not in BRCA cases that were not modified (*p* = 0.0444; Figure S2B). Patients with BRCA or UCEC who did not have their CCDC97 changed did not demonstrate substantial improvement in their OS rate (*p* = 0.0531 and *p* = 0.0631, respectively). Similarly, the SARC cases that had modified CCDC97 did not show any improvement in prognosis (Figure S2C).

### 3.4. Methylation Analysis of CCDC97 in Pan Cancer

According to the UALCAN portal, higher levels of CCDC97 expression in BLCA, HNSC, kidney renal papillary cell carcinoma (KIRP) LIHC, prostate adenocarcinoma (PRAD), testicular germ cell tumor (TGCT), THCA, and UCEC were linked to a reduction in promoter methylation (Figure 4A). The methylation level of CCDC97 in COAD, KIRC, LUSC, PAAD, and SARC was markedly elevated compared to normal tissues (Figure S3A).

Figure 4CCDC97 methylation levels in pan cancer. (A) The amount of methylation associated with the CCDC97 promoter decreased (blue is nomal and red is primary tumor). (B) The investigation examined, employing Spearman’s rho values and statistical significance, the link between CCDC97 expression and mRNA modification methylation regulatory factors was investigated.  ^∗^ *p* < 0.05;  ^∗∗^ *p* < 0.01;  ^∗∗∗^ *p* < 0.001.

**Figure:**
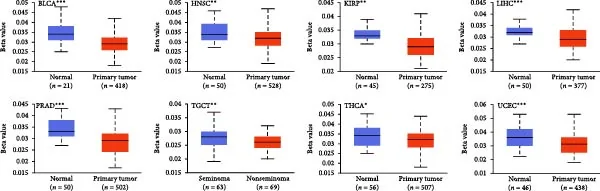
(A)

**Figure:**
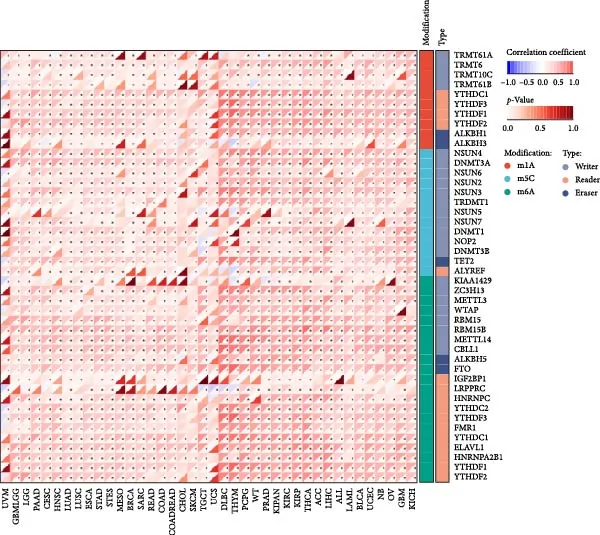
(B)

Based on the methylation map of CCDC97 from the MethSurv database, the largest amount of DNA methylation was noticed in the cg03313447 site of BLCA, HNSC, KIRP, LIHC, and UCEC (Figure S3B).

Moreover, the mRNA modification parameter is crucial in the realm of epigenetics and is intricately engaged in the control of post‐transcriptional genes. Prior research has documented have firmly shown a significant correlation of alterations in mRNA with the onset and progression of cancer [22]. Among these, m1A, m5C, and m6A stand out as the predominant types of mRNA modification. Accordingly, this study continued, with specific attention paid to methyltransferases (writers), demethylases (erasers), and RNA‐binding proteins (readers), toexamine the correlation between CCDC97 expression and 44 regulators of mRNA modification. As depicted in Figure 4B, there was a positive correlation between CCDC97 expression and the majority of m1A, m5C, and m6A methylations in pan cancer tissues.

Typically, the abnormally high synthesis of CCDC97 mRNA might be linked to decreased levels of DNA methylation.

### 3.5. Phosphorylation Modifications of CCDC97 in Pan Cancer

Protein phosphorylation is a regulatory process that controls gene expression by altering the readability of genes. Figure 5A outlines the phosphorylation sites of CCDC97 and its notable distinctions.

Figure 5Analysis of phosphorylation in the CCDC97 protein across various types of cancers. A comparison was made between the expression levels of CCDC97 phosphoprotein (NP_001333029, S147, S192, S199, S212, S257, and S272 sites) in primary tumors and normal tissues using the CPTAC dataset, which was obtained from the UALCAN. (A) Phosphorylation sites schematic design for CCDC97. (B–F) The box plots depicting various types of malignancies are presented.  ^∗^ *p* < 0.05;  ^∗∗^ *p* < 0.01;  ^∗∗∗^ *p* < 0.001 (blue is nomal and red is primary tumor).

**Figure:**
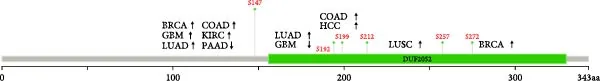
(A)

**Figure:**
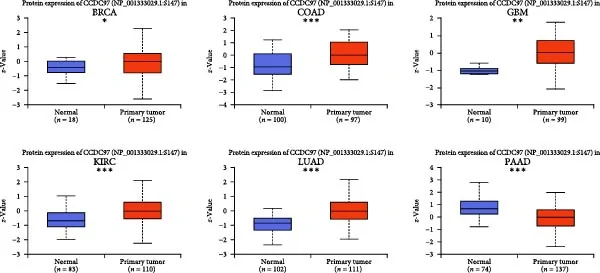
(B)

**Figure:**
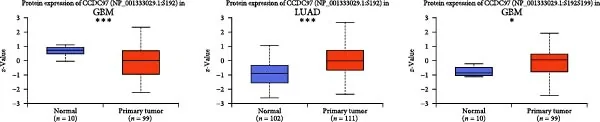
(C)

**Figure:**
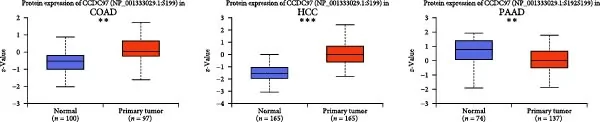
(D)

**Figure:**
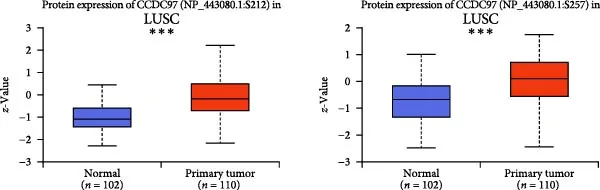
(E)

**Figure:**
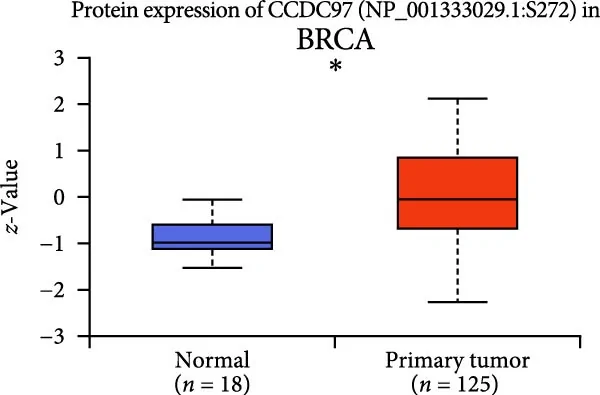
(F)

As a result, compared to normal tissues, the S147 location of CCDC97 exhibited increased phosphorylation levels in BRCA, COAD, GBM, KIRC, and LUAD. In contrast, as observed in Figure 5B solely, the phosphorylation levels in PAAD were much lower than those in normal tissues. Subsequently, the phosphorylation level of the S192 site was elevated in LUAD and reduced in GBM compared to normal tissues (Figure 5C). The S199 location within the DUF2052 domain exhibited elevated levels of phosphorylation in COAD and HCC (Figure 5D). Moreover, the phosphorylation levels of Site S212 and S257 were particularly tested in the LUSC, which were greater than those noticed in normal tissues (Figure 5E). Besides, phosphorylation alterations were observed exclusively in BRCA in the S272 (Figure 5F).

### 3.6. Relationship Between Pan Cancer CCDC97 Expression, Immunological Checkpoint, TMB, MSI, and MMRs

The immunological checkpoint mechanism enables tumor cells to evade the immune system. As a consequence of this, immune checkpoint blockade has become an important anti‐tumor strategy [23]. As shown in Figure 6A, the expression of CCDC97 revealed positive correlations with the expression of immune checkpoint markers in several cancer types, such as COAD, COADREAD, DLBC, GBMLGG, HNSC, KIRC, KIRP, LGG, LIHC, STAD, and STES.

Figure 6The correlation between the expression of CCDC97 and various immunotherapy‐related reactions. An examination of the relationship between the expression of CCDC97 and immunological checkpoints (A), the relationship between the expression of CCDC97 and MMRs (B), TMB (C), MSI (D), and NEO (E) in different types of malignancies.  ^∗^ *p* < 0.05.

**Figure:**
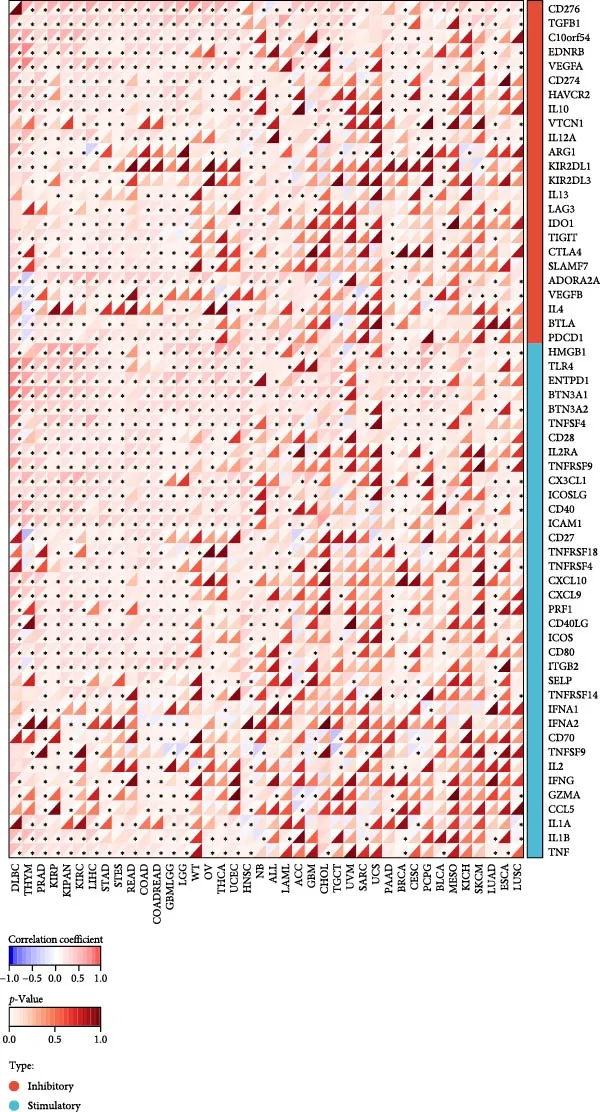
(A)

**Figure:**
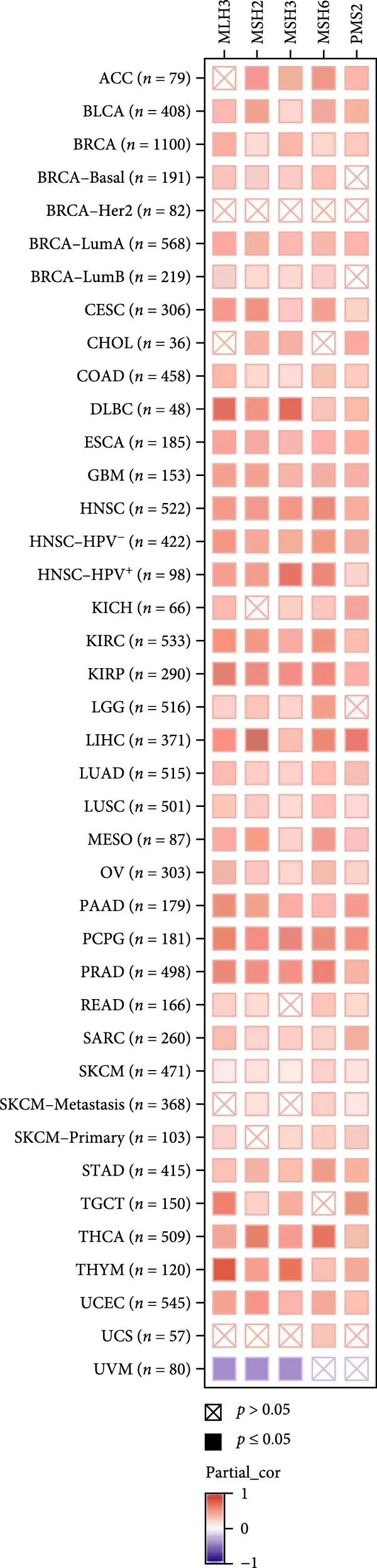
(B)

**Figure:**
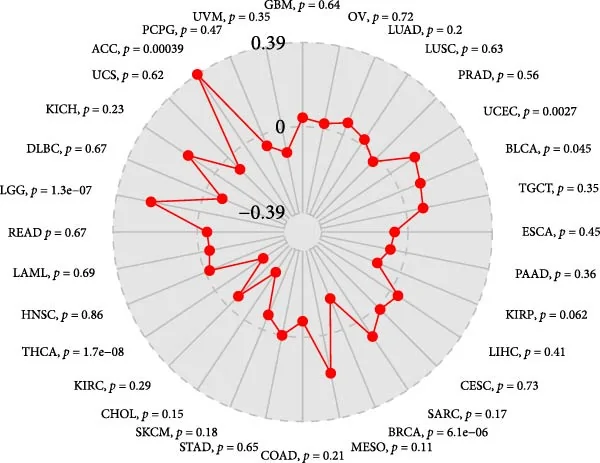
(C)

**Figure:**
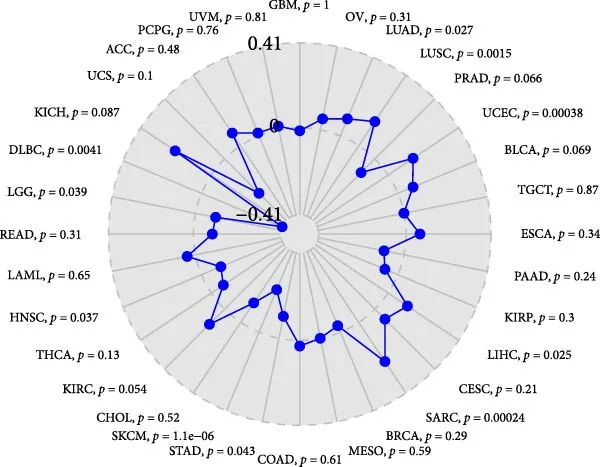
(D)

**Figure:**
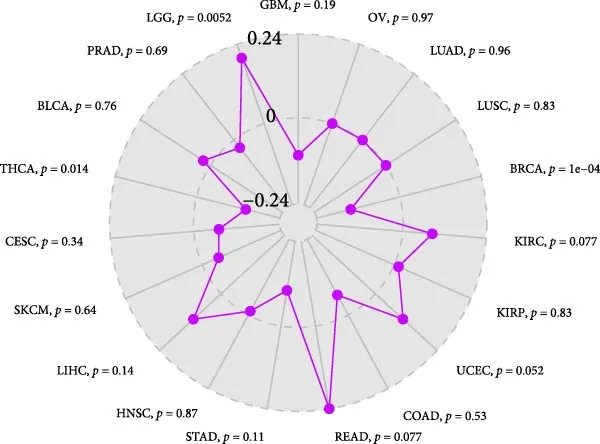
(E)

Furthermore, MMR is essential for maintaining the integrity of the genome by safeguarding it against random DNA damage [24]. Strong associations were found between CCDC97 and multiple genes related to MMR, (i.e., MLH3, MSH2, MSH3, MSH6, and PMS2), in several forms of cancer, including BLCA, BRCA, CESC, COAD, DLBC, ESCA, GBM, HNSC, KIRC, KIRP, LIHC, LUAD, LUSC, MESO, OV, PAAD, PCPG, PRAD, SARC, SKCM, STAD, THCA, THYM, and UCEC (Figure 6B).

In many types of cancer, TMB functions as an immunotherapy biomarker, enabling a prediction of the therapeutic efficacy of immune checkpoint‐targeting drugs [25]. In our study, TMB and CCDC97 expression were positively correlated in ACC, BLCA, LGG, and UCEC, while TMB was negatively correlated withCCDC97 expression in BRCA and THCA (Figure 6C).

Meanwhile, MSI with a direct association with tumor growth, can serve as an indicator of patients’ response to immunotherapy and chemotherapy [24]. MSI is a result of inadequacies in MMR. CCDC97 expression also exhibited positive associations with MSI in LIHC, LUAD, LUSC, SARC, and UCEC. Conversely, negative associations were detected between CCDC97 expression and MSI in DLBC, HNSC, LGG, STAD, and SKCM (Figure 6D).

Increased TMB and elevated MSI may lead to the production of more NEOs, supporting its potential as a biomarker in cancer immunotherapy [25, 26]. Cases of LGG were the only ones in which a positive correlation was found between CCDC97 expression and NEO, and negatively correlated withBRCA andTHCA (Figure 6E). Collectively, CCDC97 enables an accurate prediction of the efficacy of immune checkpoint inhibitors in the treatment of various cancers.

### 3.7. The Correlation Between CCDC97 Expression and Immune Infiltration in Pan Cancer

To comprehend the correlation between CCDC97 and cancer immunity, the correlation between the expression of CCDC97 and the infiltration of immune cells was examined by utilizing six advanced algorithms (TIMER, EPIC, quanTIseq, MCP‐counter, CIBERSORT, and xCell; Figure 7A–D and Figure S4 A and B). The results of our investigation into a number of cancer samples indicated a clear and positive association between CCDC97 and the presence of infiltrating inflammatory cells. Specifically, elevated levels of CCDC97 were observed to have a noteworthy association with multiple infiltrating inflammatory cells, such as cancer‐associated fibroblasts, regulatory T cells (Tregs), neutrophils, monocytes, macrophages, dendritic cells, regulatory natural killer cells, and mast cells, in the majority of cancer cases (all *p* < 0.05). Among the four algorithms of TIMER, EPIC, quanTIseq, and MCP counter, liver cancer samples showed the highest expression correlation among neutrophils, other cells, M2 macrophages, and monocytic lineage immune cells (with values of 0.43, 0.50, 0.33, and 0.32, respectively). Altogether, Among the four algorithms of TIMER, EPIC, quanTIseq, and MCP counter, liver cancer samples showed the highest expression correlation among neutrophils, other cells, M2 macrophages, and monocytic lineage immune cells (Pearson’s correlation, *p* = 0.43, 0.50, 0.33, and 0.32, respectively) the presence of CCDC97 may have an effect on the progression of cancer, as well as corresponding prognosis and treatment.

Figure 7The relationship between the expression of CCDC97 and the presence of immune cells infiltrating the tissue. The correlation between CCDC97 and immunological infiltrates was examined using the TIMER (A), EPIC (B), quanTIseq (C), and MCP‐counter (D) algorithms. The three tumors that exhibit the strongest connection between CCDC97 expression and stromal score (E), immune score (F), and estimation score (G).  ^∗^ *p* < 0.05,  ^∗∗^ *p* < 0.01,  ^∗∗∗^ *p* < 0.001,  ^∗∗∗∗^ *p* < 0.0001.

**Figure:**
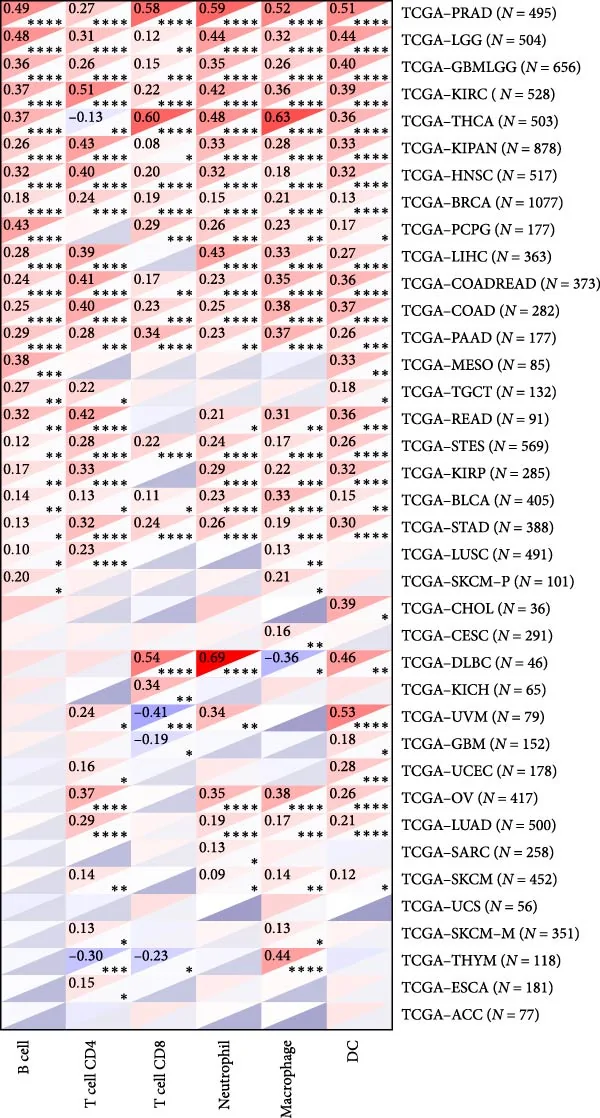
(A)

**Figure:**
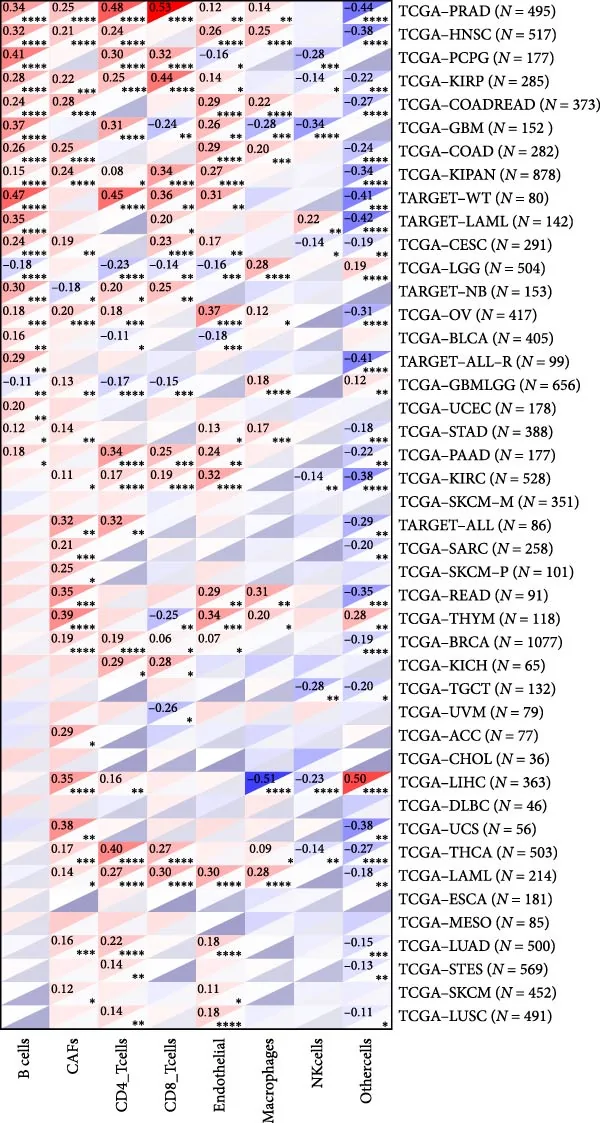
(B)

**Figure:**
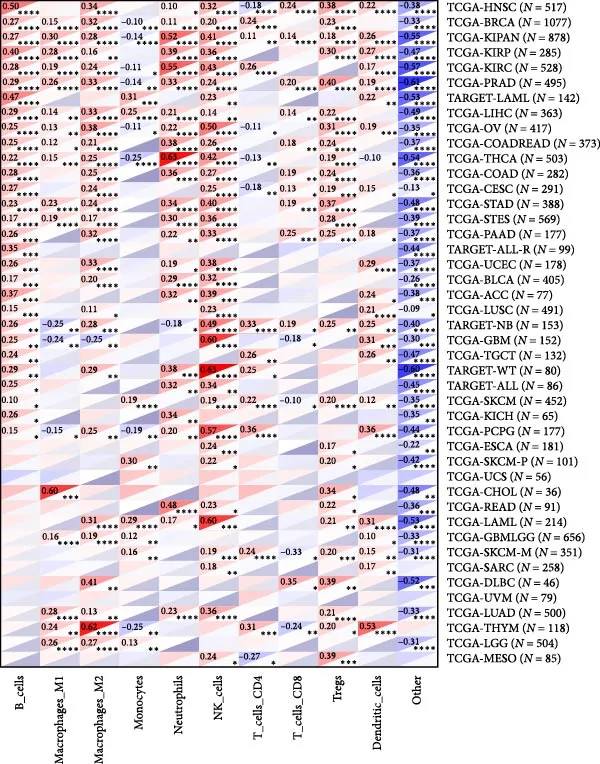
(C)

**Figure:**
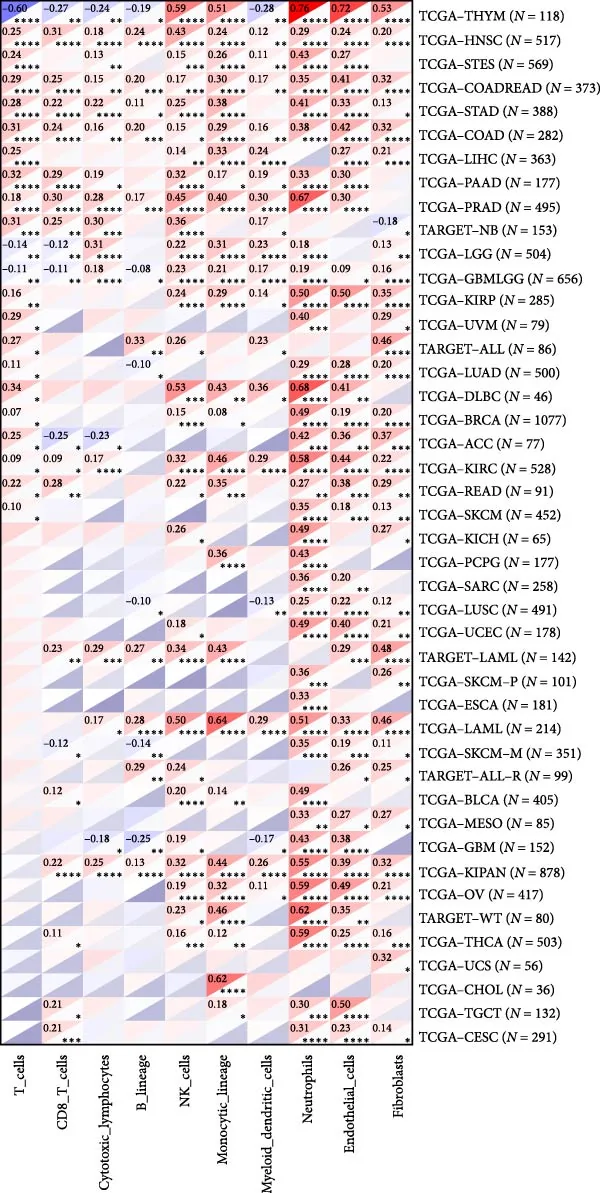
(D)

**Figure:**
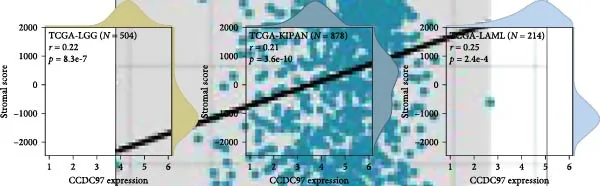
(E)

**Figure:**
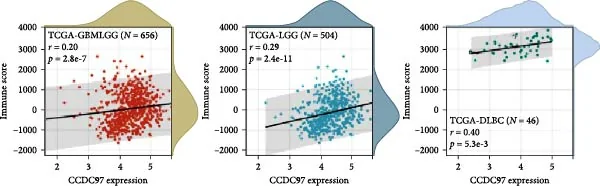
(F)

**Figure:**
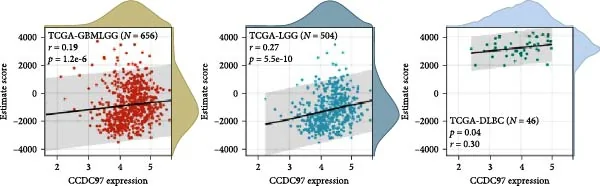
(G)

Besides, we examined the correlation between tumor purity and CCDC97 expression. The TCGA database data was incorporated to calculate the stroma, immune, and estimate scores of relevant tumor samples using the ESTIMATE tool, followed by the evaluation of the association between these ratings and CCDC97 expression. As a result, LAML, LGG, and KIPAN were the three types of cancer that had the most robust correlations between CCDC97 and the stroma score (Figure 7E). Meanwhile, the three tumor types that exhibited the most significant correlation between immunological score and CCDC97 expression were GBMLGG, LGG, and DLBC (Figure 7F). DLBC, LGG, and GBMLGG were the three tumor types with the highest association between CCDC97 and estimate scores (Figure 7G). Therefore, CCDC97 expression correlated strongly with tumor purity and immune cell infiltration into the tumor.

### 3.8. Single‐Cell Analysis of CCDC97 Expression in Pan Cancer in TME

TME analysis might be facilitated by using the extensive curated database known as TISCH2, which incorporates single‐cell transcriptome characteristics of roughly 2 million cells from 76 tumor datasets of good quality, covering 27 different forms of cancer [14].

First, TISCH2 was searched to create a graphical representation of UMAP plots and to analyze the expression patterns of CCDC97 at the level of individual cells in pan cancer samples (Figure 8A). Supplementary research was carried out to examine the localization of CCDC97 expression in different cells of the TME across multiple cancer types (Figure S5). A heatmap (Figure 8B) was plotted to examine the expression of CCDC97 at the average level across various cell types.

Figure 8Single‐cell investigation of the pan cancer feature of CCDC97 expression. (A) The single‐cell UMAP plots that can be seen are meant to show how the cells in the treatment reaction groups are distributed (left) and how much CCDC97 is expressed (right). (B) The heat map illustrates the distribution of CCDC97 expression in different cell types across various datasets with different levels of malignancy.

**Figure:**
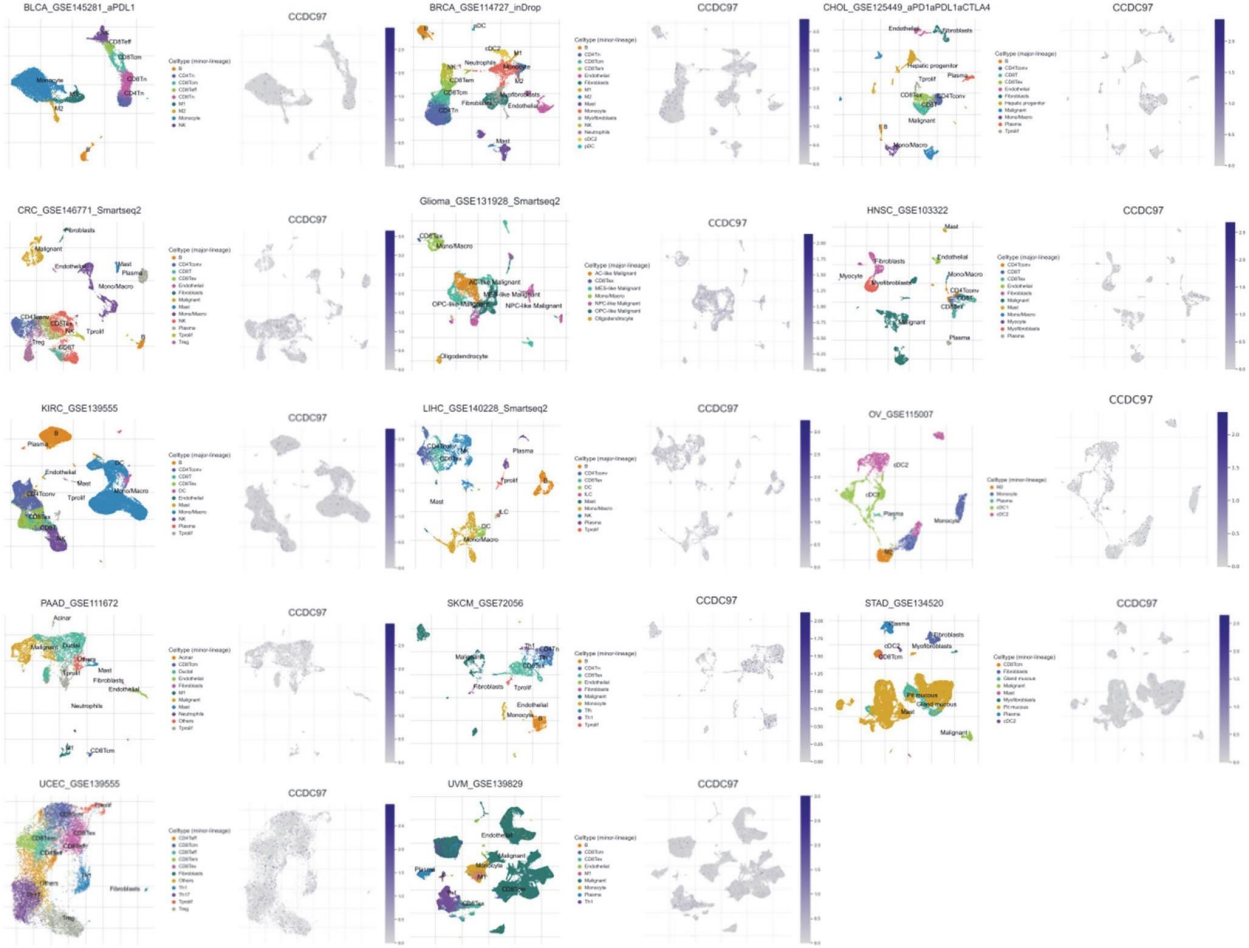
(A)

**Figure:**
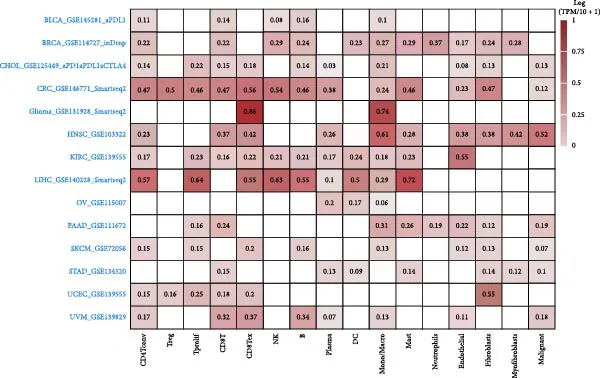
(B)

Eventually, CCDC97 expression o was significantly elevated in the TME of both CRC and HCC (Figure 8). Thus, CCDC97 exhibited significantly high expression in monocytes/macrophages and CD8^+^Tex cell types of TME (Figure 8).

### 3.9. Sensitive Drug Prediction

Based on CCDC97 levels, we discovered substances and treatments for multiple types of malignancies utilizing public datasets. Genes that were resistant or susceptible to the treatment were indicated by positive or negative correlations. The Cancer Therapeutics Response Portal dataset revealed that medication sensitivity and CCDC97 had an undesirable connection. In particular, the top‐3 medications that seriously attributable to CCDC97 expression were BRD‐K34222889, nakiterpiosin, and BRD‐K48334597 (Figure 9A).

Figure 9Prediction of pharmaceuticals based on the expression of CCDC97 in pan cancer using the CTRP (A) and GDSC (B) datasets.

**Figure:**
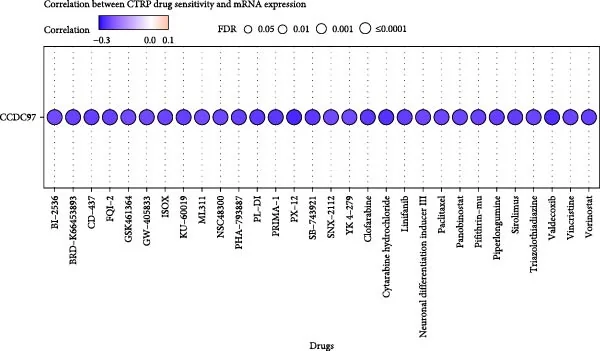
(A)

**Figure:**
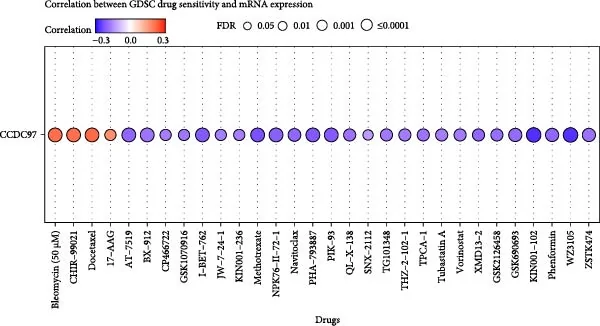
(B)

The Genomics of Drug Sensitivity in Cancer collection presents data indicating a negative correlation between drug sensitivity and CCDC97 level. WZ3105, KIN001‐102, and the treatment are the three most prominent drugs that have a negative correlation with CCDC97. Conversely, drugs with the strongest positive correlation with CCDC97 were docetaxel, bleomycin (50 μM), and CHIR‐99021 (Figure 9B).

### 3.10. Gene Enrichment Analysis of CCDC97 in Pan Cancer

In order to better understand the impact of CCDC97 gene on tumor development, an investigation was conducted to identify the particular proteins that interacted with CCDC97 and genes that were associated with CCDC97 expression. The results of these investigations were then involved in various different pathway enrichment studies. The STRING program was used to identify 41 proteins that bound to CCDC97 which was supported by evidence obtained through controlled experiments. Figure 10A illustrates the network of protein interactions.

Figure 10CCDC97‐related gene enrichment analysis. (A) Using the STRING tool to obtain the available experimentally determined CCDC97‐binding proteins. (B) The top 100 CCDC97‐correlated genes in TCGA projects and analyzed the expression correlation between CCDC97 and selected targeting genes, including HNRNPUL1, ZC3H4, ZNF526, ATP5SL, and ZNF574. (C) The heatmap data corresponding to the specific cancer types are presented. (D) An intersection analysis of the CCDC97‐binding (left) and correlated genes (right) was conducted. (E, F) Based on the CCDC97‐binding and interacted genes, GO and KEGG pathway analysis was performed.

**Figure:**
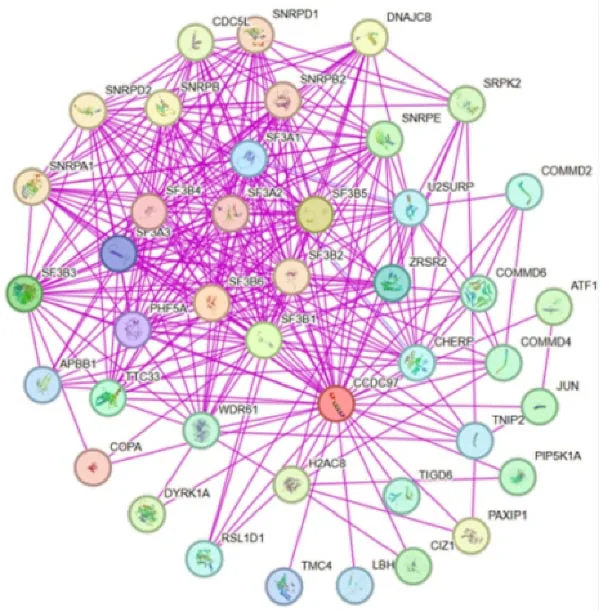
(A)

**Figure:**
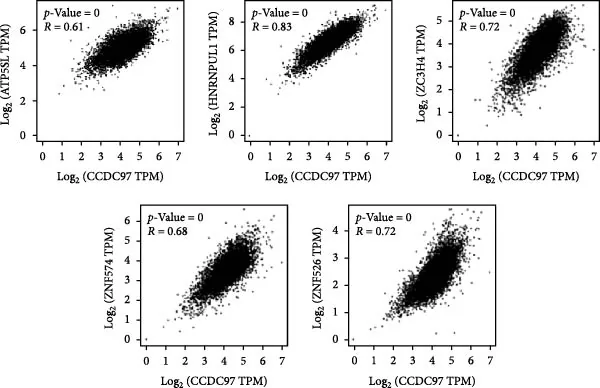
(B)

**Figure:**
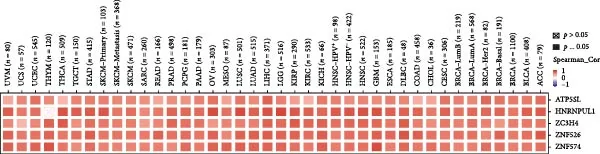
(C)

**Figure:**
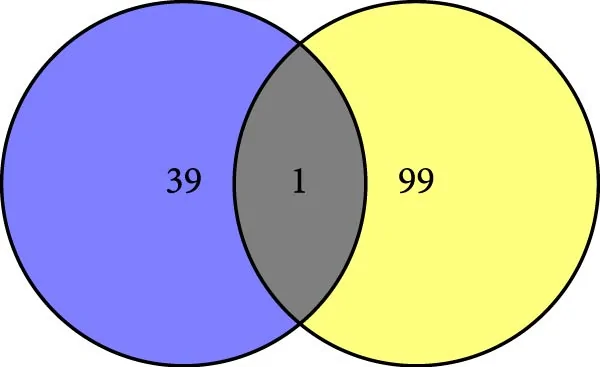
(D)

**Figure:**
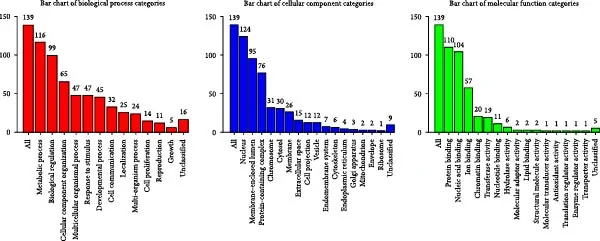
(E)

**Figure:**
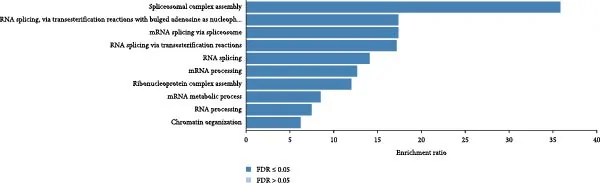
(F)

The entire TCGA tumor expression data was s based on the GEPIA2 algorithm, with an identification of the top‐100 genes that exhibited strong correlations with CCDC97 expression. As illustrated in Figure 10B, a direct relationship was observed between CCDC97 expression and the expression levels of Heterogeneous Nuclear ribonucleoprotein U Like 1 (*R* = 0.83), Zinc Finger CCCH‐Type Containing 4 (*R* = 0.72), Zinc Finger Protein 526 (*R* = 0.72), ATP Synthase Subunit S‐Like Protein 5 (*R* = 0.61) and Zinc Finger Protein 574 (*R* = 0.68; all *p* < 0.001).

As illustrated in the heatmap (Figure 10C), most of the specified cancer types also showed positive correlations between CCDC97 and the five genes mentioned above. Further intersection analysis identified that the two groups mentioned above shared one member, DYRK1A (Figure 10D).

With respect to the above, the two datasets were merged to conduct KEGG and GO enrichment analyses. According to the KEGG data, the impact of CCDC97 on tumor etiology might entail “spliceosomal complex assembly” and “mRNA splicing, via spliceosome” (Figure 10E). GO enrichment analysis indicated that the majority of these genes were primarily associated with biological regulation, protein binding, nucleic acid binding, the nucleus, membrane‐enclosed lumen, metabolic processes, and other related functions (Figure 10F).

### 3.11. Construction of CCDC97 ceRNA Regulatory Network in HCC

The ceRNA regulatory network has been shown to be a potentially valuable biomarker for HCC diagnosis and treatment clinically [27]. Thus, we endeavored to construct a ceRNA network by including the CCDC97 gene in HCC.

Venn diagrams representing the anticipated CCDC97–targeting miRNAs in LIHC were produced by the three databases starBase, miRDB, and TargetScan, which are, in order, 69, 149, and 745. Intersection of the three datasets resulted in 19 targeted miRNAs (Figure 11A). After that, to determine which targeted miRNAs were considered to be the most appropriate, we investigated the expression relationship between the 19 targeted miRNAs and CCDC97. In Figure 11B, the expression of hsa‐miR‐122‐5p (*r* = −0.438, *p* = 8.46e−19) and hsa‐miR‐22‐3p (*r* = −0.585, *p* = 2.56e−35) was inversely correlated with CCDC97 expression.

Figure 11Forecasting and detection of the CCDC97 competing endogenous RNA (ceRNA) network. (A) CCDC97‐targeting microRNAs are displayed in the miRDB, starBase, and TargetScan tools, as shown in the Venn diagram. (B) starBase was used to examine significant relationships between CCDC97 and targeted miRNAs. (C) starBase and miRNet2.0 projected the targeting‐lncRNAs of hsa‐miR‐122‐5p (C1) and 22‐3p (C2) as a Venn diagram. (D) Scatter plots showing how hsa‐miR‐122‐5p expression and the expression of DSCAM‐AS1, GUSBP11, XIST, NEAT1, SNHG11, KCNQ1OT1, and LINC00294 are closely correlated. (E) Scatter plots showing the strong relationships between the expression of MALAT1, LINC00630, OIP5‐AS1, and H19 and hsa‐miR‐22‐3p. (F) Sankey diagram showing ceRNA network’s final correlations.

**Figure:**
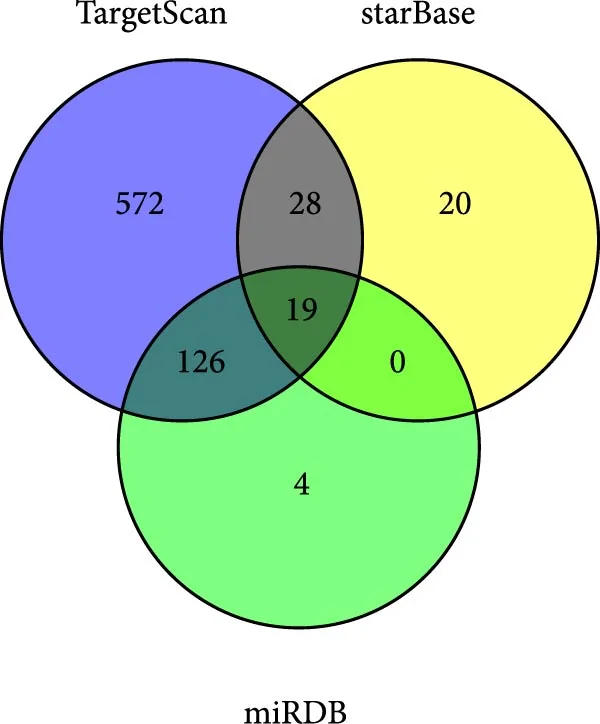
(A)

**Figure:**
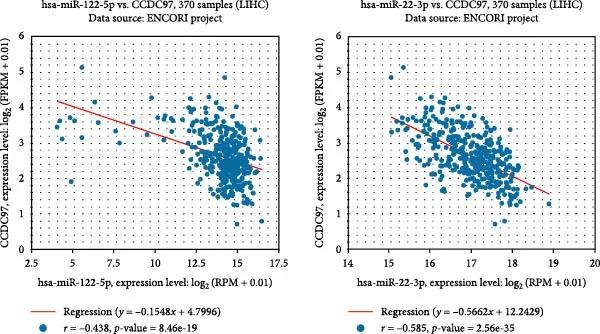
(B)

**Figure:**
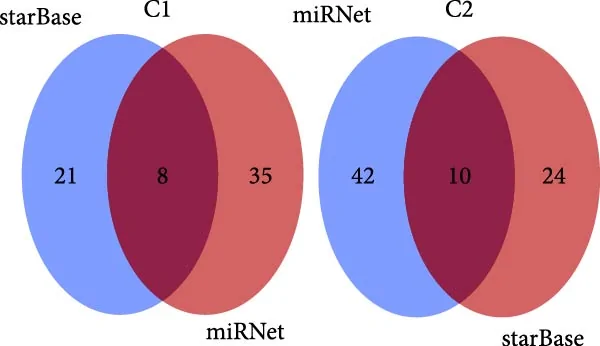
(C)

**Figure:**
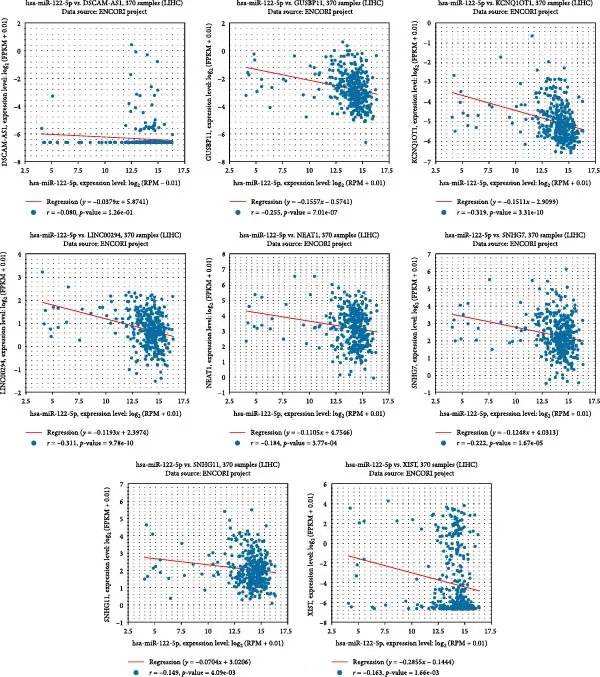
(D)

**Figure:**
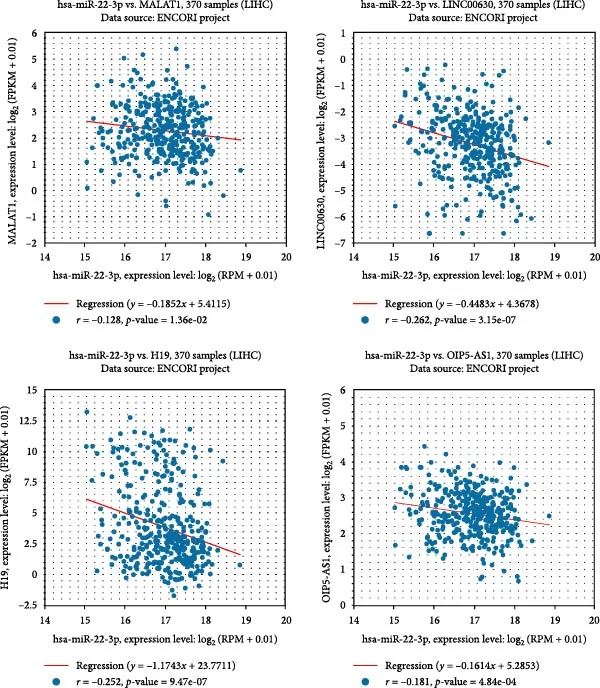
(E)

**Figure:**
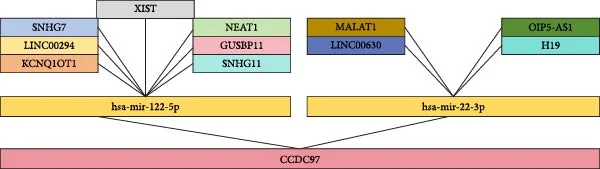
(F)

Then, the starBase and miRNet2.0 databases were employed to predict the targeting‐lncRNA of hsa‐miR‐122‐5p and hsa‐miR‐22‐3p in LIHC via a Venn diagram. Intersection of the results from starBase and miRNet2.0 yielded 10 hsa‐miR‐22‐3p‐lncRNAs (SNHG16, NEAT1, NORAD, FGD5‐AS1, GUSBP11, MALAT1, LINC00630, OIP5‐AS1, H19, and LINC00963) and eight hsa‐miR‐122‐5p‐lncRNAs (SNHG7, KCNQ1OT1, LINC00294, NEAT1, SNHG11, DSCAM‐AS1, GUSBP11, and XIST; Figure 11C).

Afterwards, the starBase database was also utilized to examine whether there was a negative expression association between these lncRNAs and the matching miRNA. As presented in in Figure 11D hsa‐miR‐122‐5p was negatively correlated with the following expression levels: SNHG7 (*r* = −0.222, *p* = 1.67e−05), KCNQ1OT1 (*r* = −0.319, *p* = 3.31e−10), LINC00294 (*r* = −0.311, *p* = 9.78e−10), NEAT1 (*r* = −0.184, *p* = 3.77e−04), SNHG11 (*r* = −0.149, *p* = 4.09e−03), DSCAM‐AS1 (*r* = −0.080, *p* = 1.26e−01), GUSBP11 (*r* = −0.255, *p* = 7.01e−07), and XIST (*r* = −0.163, *p* = 1.66e−03). Meanwhile, in Figure 11E, hsa‐miR‐22‐3p was negatively correlated with MALAT1 (*r* = −0.128, *p* = 1.36e−02), LINC00630 (*r* = −0.262, *p* = 3.15e−07), OIP5‐AS1 (*r* = −0.181, *p* = 4.84e−04), and H19 (*r* = −0.252, *p* = 9.47e−07).

Consequently, we constructed 11 categories of ceRNA regulatory networks according to the ceRNA prediction depicted in Figure 11F.

## 4. Discussion

By utilizing TCGA, CPTAC, and GTEx databases, this study investigated the mRNA and antigen production levels of CCDC97 in both cancerous and typical tissues. As evidenced previously, CCDC97 is elevated in breast invasive carcinoma when compared to that in nontumor tissues [28]. In our study, CCDC97 was correlated with upregulated in most cancers. Moreover, increased expression of CCDC97 was correlated with the poor prognosis of patients diagnosed with ACC, BLCA, LGG, and HCC. In contrast, CCDC97 expressed lowly in patients diagnosed with clear cell renal cell carcinoma, which had a correlation with poor prognosis. Additionally, simultaneous expression of CCDC97 is correlated with a reduced OS and the DFS rates in triple‐negative breast cancer (BRCA) patients [28]. DYRK1B can also enhance the Hedgehog signaling pathway [29], which is essential for the development of cancer and stemness [30]. At this stage, further exploration is required to unveil similar or other underlying mechanisms in additional tumors.

Furthermore, it has been documented that the degree of DNA methylation presented in the promoter region of the CCDC97 gene in 13 different types of cancer and normal tissues and indicated a significant inhibition of gene expression due to methylation of the promoter region [31]. Several categories of cancers, including BLCA, HNSC, KIRP, HCC, PRAD, TGCTs, THCA, and UCEC, exhibit hypomethylated versions of the CCDC97 gene. BLCA, HNSC, HCC, and UCEC were discovered to possess nine CpG sites in CCDC97. In our study, CCDC97 was relevant to the majority of m1A, m5C, and m6A methylation regulators in various cancers. Therefore, CCDC97 could be employed as a prospective diagnostic marker to identify mutations and pan cancer epigenetic modifications.

In most cancers (e.g., BRCA, COAD, GBM, KIRC, and LUAD), CCDC97 phosphorylation was significantly increased at Ser147. Despite unclear understanding of the functional consequence of CCDC97 phosphorylation CCDC106 from the CCDC family has been proven to promote the development of BRCA and cervical cancer by inhibiting apoptosis. Furthermore, CCDC106 can interact with p53 and its oncogenic function depends on the phosphorylation of Ser147 by CK2 [32]. On the above basis, we speculate that the phosphorylation of CCDC97 at S147 may exert a similar mechanism in tumorigenesis.

Currently, it is known that immune regulatory cells in the TME can promote or inhibit tumor growth and affect the efficacy of anti‐tumor immunotherapy [33]. CD8 T cells are crucial for sustained antigen‐stimulated anti‐tumor immunity in the TME, which, however, may lose their anti‐tumor activity when they become fatigued. These cells are biologically distinct from effector cells and memory T cells, a concept proposed for the first time by Zajac and Gallimore in 1998 [34–36]. By detecting the expression of CCDC97 in pan cancer cells at single‐cell level, we found that CCDC97 was highly expressed in monocytes/macrophages and CD8 Tex.

There are complex interactions between immune regulatory cells and cytokines. Depletion of CD8^+^ T cells may be affected by immune regulatory cells and cytokine mediators, while Tregs can inhibit the differentiation and activation of CD8 cytotoxic T cells and CD4 helper T cells [37]. Accumulated evidence suggests that M2 macrophages and myeloid‐derived suppressor cells (MDSCs) can promote Treg recruitment while hindering T cell development and function, which can further boost the negative regulatory effect of Treg on immunity [38–40]. TGF‐*β*, IL‐10, and IL‐35 are cytokines secreted by M2 macrophages and Tregs, which can impair antitumor immunity, thereby accelerating cancer development and spread [41, 42]. IL‐10 and enhanced TGF‐*β* signaling have been demonstrated to contribute CD8 T cell depletion [43–45], which has been proven in BRCA and SKCM [46, 47]. In addition, M2 macrophages express several CD8^+^ T cell inhibitory receptor ligands (e.g., PD1, CTLA‐4, TIGIT, and TIM‐3). In order to promote polarization of M2 macrophages, CD8 T cells with terminal fatigue also produce related factors (MIF and CSF1) [48]. MDSCs strongly suppress the immune response of CD8 T cell–targeting tumors [49]. TIM‐3 bound to the surface of CD8 T cells is an inhibitory receptor upregulated by Gal9 binding in MDSCs, which can lead to depletion of CD8 T cells [50].

In view of these interpretations, the interaction between these immune regulatory cells and cytokine mediators may lead to the emergence of exhausted CD8^+^T cells.

In our study, CCDC97 was discovered to exhibited close correlations with immune regulatory cells (e.g., Tregs, M2 macrophages, and MDSCs) through the use of six latest algorithms (i.e., TIMER, EPIC, quanTIseq, MCP counter, CIBERSORT, and xCell) to quantify immune cells. Consequently, our study confirmed a correlation between CCDC97 and CD8^+^ T cells with immune regulatory cell depletion in the TME, and validated the high expression of CCDC97 in monocytes/macrophages and the aforementioned CD8^+^ T cells.

In addition, markers for the expression of CD8 Tex inhibitory receptors have been identified, including PD1, CTLA4, TIGIT, and TIM3 [51, 52]. In our research, a significant correlation was also observed between immune suppression markers expressed in immune cells (e.g., PD‐1, CTLA4, HAVCR2 [TIM‐3], and TIGIT), and high expression of CCDC97. The accumulation of Tregs, TAMs, and MDSCs, as well as T cell fatigue and PD‐L1 overexpression, are all mechanisms explaining that CCDC97 may be involved in mediating immune suppression in the TME. The expression of mRNA is another hot spot in malignant tumor research, where the competitive interaction between lncRNA and miRNA affects the expression of mRNA. For instance, the lncRNA–miRNA–mRNA regulatory pathway is clearly visible in LIHC. has‐miR‐12‐5p can prevent epithelial–mesenchymal transition (EMT) in HCC by inhibiting the WNT/*β*‐cadherin signaling pathway as well as targeting Snail1 and Snail2 [53]. hsa‐miR‐22‐3p has been reported to be responsible for indirect regulation of SPRY2, which can prevent EMT and cancer cell migration/invasion. hsa‐miR‐22‐3p is another biomarker that can indirectly regulate SPRY2, which prevents EMT and migration/invasion of HCC cells [54]. In HCC, SNHG11 stimulate cell migration and proliferation [55]. SNHG7 is an common key oncogenic lncRNA in cancer, which can promote HCC progression by downregulating the expression of has‐miR‐122‐5p [56, 57]. Along with SNHG7, KCNQ1OT1, LINC00294, NEAT1, SNHG11, GUSBP11, and XIST, investigation on has‐miR‐122‐5p may lay a the foundation for creating the lncRNA‐miRN‐CDC97 network.

In summary, this pan cancer demonstrates the potential of CCDC97 as a predictive biomarker for cancer patients, and it can effectively predict patients’ response to immunotherapy. Findings in our study may facilitate our more comprehensive understanding of the potential involvement of CCDC97 in pan cancer.

It should be acknowledged that the results of this study were generated based on bioinformatics analysis of public databases without further validation in our own experiments. However, this study comprehensively explores the potential mechanism of aCCDC97 in human cancers and also supports the results of other experimental studies. In the future, we will continue to validate experimentally in independent samples to provide more valuable insights.

## 5. Conclusions

To sum‐up, CCDC97 can serve as a promising marker for determining cancer immunotherapy outcome and prognosis, particularly in HCC. More experimental verification is needed in the future research.

## Ethics Statement

No human or animal experiments were involved in this study. This study was conducted in accordance with the guidelines of the Declaration of Helsinki. All treatment plans applied in this study were conducted in accordance with the standards of the Ethics Committee of Huishan District People’s Hospital of Wuxi City, China (Ethics Approval Code: HYLL20240712001).

## Disclosure

All the authors have approved the manuscript preparation and submission.

## Conflicts of Interest

The authors declare no conflicts of interest.

## Author Contributions

**Ruirui Yang:** data curation, formal analysis, writing – review. **Changye Yang:** investigation, data curation, formal analysis, writing – original draft. **Rundong Wang:** investigation, formal analysis. **Guo Ma**: validation, writing – review and editing. **Lixin Hua:** conceptualization,validation, writing – review and editing. Changye Yang and Ruirui Yang contributed equally to this work and shared first authorship.

## Funding

This study was supported by the Clinical Medicine Special Research Fund Project of the Nantong University (Key Project, Grant 2024JZ044), the Natural Science Foundation Project of Shandong Province (Grant ZR2020MC073), and the University Student Innovation and Entrepreneurship Program (Grant S202010439049).

## Supporting Information

Additional supporting information can be found online in the Supporting Information section.

## Supporting information


**Supporting Information** Figure S1. Expression level of CCDC97 gene in different tumors and pathological stages. (A) No significant differences were found from normal tissue and tumor tissues. (B) Correlations between CCDC97 expression and pathological stage. Figure S2. Analyses of the potential correlation between mutation status and disease‐free survival (A), disease‐specific survival (B), and overall survival (C) of BRCA, SARC, UCEC, and UCS. Figure S3. The methylation levels of CCDC97 in pan cancer. (A) The promoter methylation level of CCDC97 was increased. (B) The heatmap of CCDC97 DNA methylation in from MethSurv.  ^∗^ *p* < 0.05;  ^∗∗^ *p* < 0.01;  ^∗∗∗^ *p* < 0.001. Figure S4. Correlation between CCDC97 and immune infiltrates analyzed by the CIBERSORT (A) and xCELL (B)  ^∗^ *p* < 0.05. Figure S5. The grid violin plot reflects the distribution of CCDC97 expression in different cell types across all datasets with various cancers.

## Data Availability

The datasets generated and/or analyzed during the current study are available in the repository. All original datasets were sourced from the following resources in the public domain. 1. TIMER2.0—http://timer.cistrome.org/ [8]. 2. GEPIA2—http://gepia2.cancer-pku.cn/ [9]. 3. UALCAN—http://ualcan.path.uab.edu/index.html [10]. 4. cBioPortal—https://www.cbioportal.org/ [11]. 5. MethSurv—https://biit.cs.ut.ee/methsurv/ [12]. 6. SangerBox 3.0—http://sangerbox.com/ [13]. 7. TISCH2—http://tisch.comp-genomics.org/home/ [14]. 8. GSCALite—http://bioinfo.life.hust.edu.cn/web/GSCALite/ [15]. 9. STRING—https://string-db.org/ [16]. 10. WebGestalt—http://www.webgestalt.org [17]. 11. miRWalk—http://mirwalk.umm.uni-heidelberg.de/ [18]. 12. miRDB—http://mirdb.org/ [19]. 13. TargetScan—https://www.targetscan.org/vert_80/ [20]. 14. starBase v2.0—https://starbase.sysu.edu.cn/ [21].
