# Supplementary material for: CCDC97 is a Promising Predictor in Cancer Diagnosis: A Pan Cancer Analysis
Source: Anal Cell Pathol (Amst). 2026 Jan 8;2026:2230769. doi: 10.1155/ancp/2230769 (PMC13580819; doi:10.1155/ancp/2230769)
Supplement: Supplementary file 1 [file ANCP-2026-2230769-s001.docx]

Supplementary Materials

**Figure Legends**

**Supplementary Figure 1.** Expression level of CCDC97 gene in different tumors and pathological stages. (A) No significant differences were found from normal tissue and tumor tissues. (B) Correlations between CCDC97 expression and pathological stage.

**Supplementary Figure 2.** Analyses of the potential correlation between mutation status and Disease-free survival(A), Disease-specific survival(B) and Overall survival(C) of BRCA, SARC, UCEC and UCS.

**Supplementary Figure 3. The methylation levels of CCDC97 in pan-cancer.** (A) The promoter methylation level of CCDC97 was increased. (B) The heatmap of CCDC97 DNA methylation in from MethSurv. *P < 0.05; **P < 0.01; ***P < 0.001.

**Supplementary Figure 4.** Correlation between CCDC97 and immune infiltrates analyzed by the CIBERSORT (A) and xCELL (B) *p < 0.05.

**Supplementary Figure 5.** The grid violin plot reflects the distribution of CCDC97 expression in different cell types across all datasets with various cancers.

**Supplementary Figure 1**

**A**

**B**

**Supplementary Figure 2**

**A**

**B**

**C**

**Supplementary Figure 3.**

**A**

**B**

**Supplementary Figure 4**

| **A**   | **B**   |
| --- | --- |

**Supplementary Figure 5**

|  |  |
| --- | --- |
